# Organic Farming and Landscape Structure: Effects on Insect-Pollinated Plant Diversity in Intensively Managed Grasslands

**DOI:** 10.1371/journal.pone.0038073

**Published:** 2012-05-30

**Authors:** Eileen F. Power, Daniel L. Kelly, Jane C. Stout

**Affiliations:** 1 Botany Department, School of Natural Sciences, Trinity College Dublin, Dublin, Republic of Ireland; 2 Trinity Centre for Biodiversity Research, Trinity College Dublin, Dublin, Republic of Ireland; University of Northampton, United Kingdom

## Abstract

Parallel declines in insect-pollinated plants and their pollinators have been reported as a result of agricultural intensification. Intensive arable plant communities have previously been shown to contain higher proportions of self-pollinated plants compared to natural or semi-natural plant communities. Though intensive grasslands are widespread, it is not known whether they show similar patterns to arable systems nor whether local and/or landscape factors are influential. We investigated plant community composition in 10 pairs of organic and conventional dairy farms across Ireland in relation to the local and landscape context. Relationships between plant groups and local factors (farming system, position in field and soil parameters) and landscape factors (e.g. landscape complexity) were investigated. The percentage cover of unimproved grassland was used as an inverse predictor of landscape complexity, as it was negatively correlated with habitat-type diversity. Intensive grasslands (organic and conventional) contained more insect-pollinated forbs than non-insect pollinated forbs. Organic field centres contained more insect-pollinated forbs than conventional field centres. Insect-pollinated forb richness in field edges (but not field centres) increased with increasing landscape complexity (% unimproved grassland) within 1, 3, 4 and 5****km radii around sites, whereas non-insect pollinated forb richness was unrelated to landscape complexity. Pollination systems within intensive grassland communities may be different from those in arable systems. Our results indicate that organic management increases plant richness in field centres, but that landscape complexity exerts strong influences in both organic and conventional field edges. Insect-pollinated forb richness, unlike that for non-insect pollinated forbs, showed positive relationships to landscape complexity reflecting what has been documented for bees and other pollinators. The insect-pollinated forbs, their pollinators and landscape context are clearly linked. This needs to be taken into account when managing and conserving insect-pollinated plant and pollinator communities.

## Introduction

Animal-mediated pollination is required for successful reproduction in many angiosperms [1]. However, there are concerns for the future of many pollinator species due to agricultural intensification [2]. Parallel declines in insect-pollinated plants and their pollinators have been reported [3]. Changes in plant communities in intensively managed arable systems are evident, with a dominance of self-pollinated plants that can better withstand disturbance (frequent soil cultivation, crop harvesting and crop rotations) over plants that depend on animals for pollination and/or other plants as pollen donors [4], [5]. In comparison, natural or semi-natural systems tend to have lower proportions of self-pollinated plants [6].

It is not clear whether intensive grasslands also have reduced proportions of insect-pollinated plants. Most western European lowland grasslands - covering millions of hectares - are intensively managed [7]. Intensive grasslands receive high fertilizer application rates and frequent defoliation [8]. This results in degraded species pools and structurally homogenous swards. The majority of agricultural land in the Republic of Ireland is intensive grassland [9]; these intensive grasslands support considerably fewer plant [10] and pollinator [11], [12] species compared to the communities of plants [13], [14] and pollinators [15], [16] found in semi-natural grasslands. Declines in plant species richness are likely to be reflected in declines in insect-pollinated plant numbers within intensive grassland plant communities. Declines in insect-pollinated plant numbers may, in turn, have further knock-on impacts on pollinators and plant-pollinator interaction networks.

Local factors such as abiotic conditions (e.g. nutrient availability and soil acidity) and field management (e.g. herbicide application and defoliation) can affect the distribution and species richness of arable plants [17], [18]. In particular, organic farming has been found to mitigate the decline of plants and pollinators in arable systems [19], [20], most likely through the prohibition of pesticides and chemical fertilizers (European Union Regulation 2092/91/EEC). There is also evidence of a shift to more insect-pollinated plants in organically-farmed arable weed communities compared to their conventional counterparts, probably as a result of increases in both plant and pollinator diversity on organic farms [5], [21]. This has important implications for organic intensive grasslands (characterised by lower stocking densities and no chemical fertilisers or pesticides), where increased plant and pollinator diversities have also been observed [12], [22].

Landscape structure, calibrated in terms of landscape complexity and the proportion of the landscape that is organically farmed, has been found to affect the influence of organic farming on local plant and pollinator diversity [23], [24], [25]. Therefore, as plants and pollinators can be affected by landscape structure and pollinator diversity can decline in parallel with insect-pollinated plants [3], one might expect insect-pollinated plant diversity to be more affected by landscape structure than non-insect pollinated plant diversity. Landscape structure, calibrated in terms of hedgerow structure and distribution, may also influence insect-pollinated plant richness, as it is clear that linear features influence pollinator movements [26], [27] and are important as food and nesting resources [16], [28], [29], [30]. Similarly, the proximity of suitable habitats/resources nearby may have a strong influence on the local distribution of pollinators and the plants they pollinate [31]. Thus, unravelling the effects of farming system and landscape structure on insect-pollinated plants may help to inform management decisions pertaining to plant and pollinator conservation.

We hypothesised that, in landscapes dominated by conventional farming, (1) plant species richness is higher in organic than in conventional dairy farms. Specifically, we expected there to be (2) a greater number of insect-pollinated forb species in organic farms than in conventional. We also expected (3) that plant community composition would differ depending on position within the field (edge vs. centre). We further hypothesised that (4) the numbers of insect-pollinated and non-insect pollinated forbs would differ from each other depending on local factors (position in field and soil parameters ) and also (5) on landscape factors (landscape complexity, presence of linear landscape features and distance metrics, i.e. proximity to different habitats). We expected that plant species richness (especially of insect-pollinated forbs) would be affected by (6) the interactions between local factors (especially local farm management) and landscape complexity. Overall, we aimed to evaluate how far grouping plants according to their principal pollen vector is useful in developing our understanding of plant relationships with local and landscape factors.

## Methods

### Ethics Statement

Permission to access sites, survey vegetation and take soil samples was obtained from all landowners.

### Study Sites

**Figure 1:**
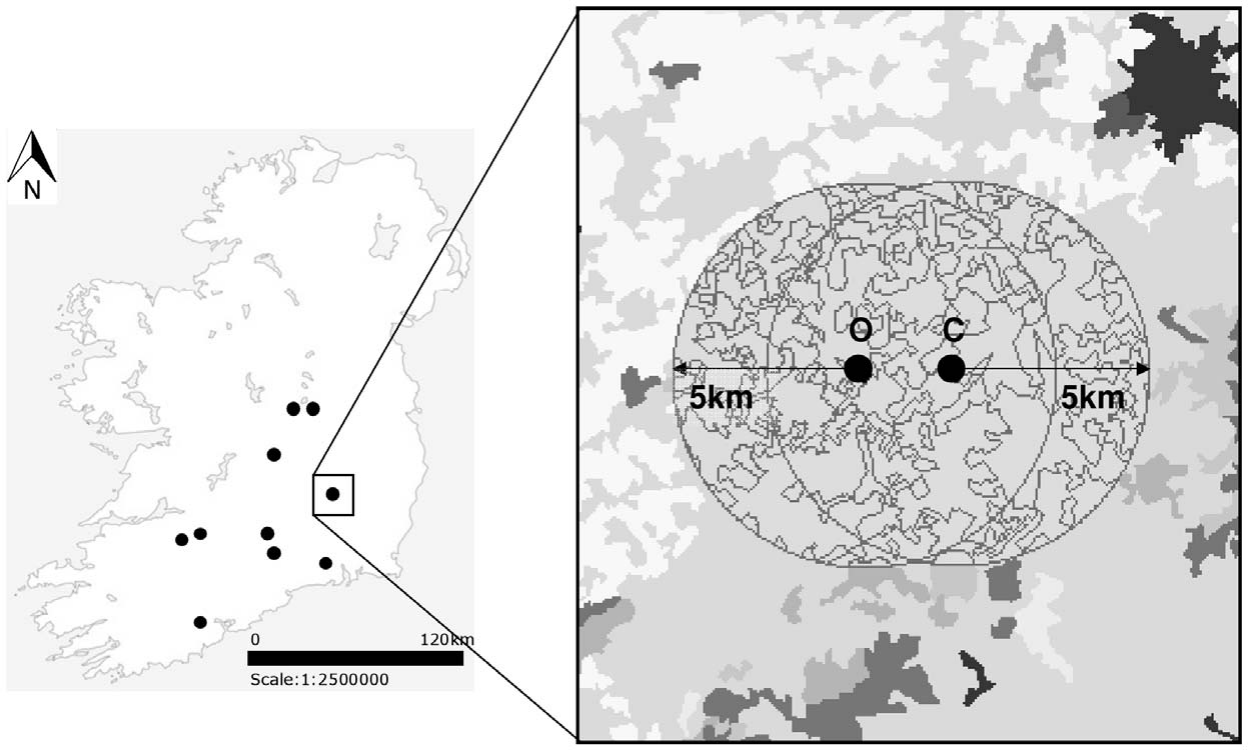
The distribution of the ten farm pairs within Ireland. For each farm, the percentage of different land-use types within 1, 2, 3, 4 and 5****km radii around farms was determined (the extent of the 5****km radii is shown as an example in this figure). Landscapes were similar and overlapping within a farm pair only. Note: CORINE Level 4 classification is displayed within the circular sectors while CORINE Level 3 classification is displayed outside them. O  =  organic farm, C  =  conventional farm.

Ten matched pairs of organic (managed according to the European Union Regulation 2092/91/EEC) and conventional (not managed according to organic regulations) dairy farms were selected in lowland permanent grassland (not ploughed or reseeded for at least 7 years) in the Republic of Ireland. Only dairy farms were chosen as effects of dairy versus drystock farming on biodiversity can differ substantially [10]. Organic farms had been certified for 10.5 years on average (range: 6–19 years), following a 2 year conversion period. Pairs were matched on geology, soil type and climatic similarity. Farms within a pair were 1–4****km apart, in landscapes dominated by conventional farming in central and southern Ireland (Fig. 1). All were in areas characterised by well-drained, fertile soils used predominantly for beef/dairy farming. The majority of organic and conventional farmers (except one conventional farmer) participated in an Irish agri-environmental scheme (the Rural Environmental Protection Scheme, REPS). Organic and conventional farms differed in terms of stocking density (average Livestock Units per hectare (LU/ha) on organic farms was 1.5 LU/ha compared with 2.5 LU/ha on conventional farms) and farm inputs. All conventional farmers in the study applied chemical fertilisers and herbicides in their fields, whilst organic farmers did not [12]. The dominant land-uses within 5****km radii around all study sites were: unimproved grassland (mean: 41%, range: 24–58%); improved grassland (mean: 35%, range: 18–46%); arable land (mean: 10%, range: 0–23%); woodland (mean: 5%, range: 0–19%); wetland (mean: 2%, range: 0–12%) and urban areas (mean: 1%, range: 0–9%).

### Plant Surveys

We surveyed forbs (broadleaved herbaceous vascular plants) and graminoids (grasses, sedges and rushes) once in three fields from each farm between July and September 2008. Fields within each farm were sampled on the same day and farms within a pair were sampled on consecutive days. Similar field sizes were surveyed between farm types (mean organic field size  = 3.2 hectares; mean conventional field size  = 3.1 hectares). Only fields that were grazed within one week of surveying were sampled. Plant surveys were conducted in two 100****m transects per field: one transect located 1****m from and parallel to the base of an internal field boundary (“field edge”) and the second transect located parallel to the first, 25****m from the same field boundary (“field centre”). The start of all edge transects were placed along stock-proof hedgerows, at least 100****m from field corners. The percentage cover of each species was recorded in five quadrats (0.5×0.5****m) placed 20****m apart in each transect, giving a total of 15 edge and 15 centre quadrats per farm. Quadrat number and size were deemed sufficient to capture the majority of plant species present by plotting species accumulation curves. For identification and nomenclature we follow Stace [32]. The forbs were classified into two groups based on their pollination types: insect and non-insect (self and/or wind-pollinated) by selecting the most frequent pollen vector according to Grime et al. [33] and the BIOFLOR database [34]. Graminoids at our sites were all wind pollinated [35] but were placed in a separate group to non-insect pollinated forbs in light of the unknown origins of many of the species (natural colonisation or planted as part of a seed mix).

### Soil Analysis

Five ca 500 g soil samples were taken along each transect, 20****m apart and from approximately 0–10 cm under the soil surface. The five soil samples from each transect were then homogenised into one sample and, for each transect sample, the pH and the available macro-nutrients Phosphorus (P), Potassium (K) and Magnesium (Mg) were analysed following Alexander et al. [36] (Table S1).

As some soil parameters were collinear, they could not all be used in the same statistical model. Therefore, we determined the relationships among the soil parameters using principal components analysis (PCA) in Primer 6.1.13 [37]. K was correlated with the first principal component (PC1), P and Mg with PC2 and to a lesser extent PC3 and pH with PC4 (Table 1). Therefore, PC1, PC2 (both representing different plant macronutrients) and PC4 (representing soil acidity) were used as covariates in further analysis.

**Table 1:** Eigenvalues for the first four principal components (PC1-4) from a principal component analysis (PCA) on soil nutrients and pH and the Pearson correlation coefficient (r) between soil parameters and the four principal components.

		Nutrient			
Principal component	Eigenvalue	P	K	Mg	pH
PC1	15.700	−0.120	−0.991***	−0.066	−0.003
PC2	0.470	−0.910***	0.136	−0.385***	−0.076
PC3	0.114	−0.386***	−0.015	0.920***	−0.069
PC4	0.009	−0.096	0.007	0.034	0.995***

Note: ***P<0.001.

### Landscape Characterisation

We used the CORINE Land Cover 2000 [38] database (100 m^2^ resolution) to characterise the landscape context of each site. This database was chosen over a more up to date version (CORINE Land Cover 2006 [39]) as it contained the level of detail required for this study (specifically, Level 4 classification which characterises pastures further into unimproved and improved grassland). The CORINE land cover information was visually compared for each site with aerial photographs (year 2005; Irish National Biodiversity Data Centre) and the CORINE Land Cover 2006 [39] database so that major changes in land cover could be identified. We discerned no major differences in Level 1 and 3 habitat classifications [38] between the databases and the aerial photographs. Difficulty in the visual identification of Level 4 classifications (unimproved and improved grassland) in the aerial photographs and the lack of this data in the CORINE Land Cover 2006 [39] database meant that the CORINE 2000 database was relied upon, in this respect.

Fifteen land use types were defined [using CORINE land cover classifications (Levels 1, 3 and 4)]: arable, unimproved grassland (medium management intensity pasture), improved grassland (high management intensity pasture), natural grassland (a low management intensity semi-natural grassland - of which there were negligible amounts recorded in the study landscapes), broadleaved forest, coniferous forest, mixed forest, transitional woodland scrub, urban areas, bogs, marshes, heaths, stream courses, water bodies and other habitats. Specific CORINE definitions of how unimproved and improved grassland are characterised are unavailable but these categories roughly correspond to semi-improved and improved grassland, respectively, in the classification of Sullivan et al. [40]. The unimproved grasslands referred to in our study deviate markedly from semi-natural grassland conditions.

The percent land cover of each of the 15 land use types was measured at five spatial scales, using 1–5****km radii around each study site (Fig. 1). Landscapes were similar around each organic and conventional farm within a pair (Figure S1) (as the spatial radii overlapped within pairs only) but not between pairs. The 1–5 km scales were chosen to reflect the potential dispersal distances of pollinators upon whom insect-pollinated forbs depend. Studies on bumblebees and honeybees have found that they can be related to the landscape at scales of up to 3 km radii [41], [42] while hoverflies can be related to the landscape up to 4****km around study sites [43] and disperse up to 400****m in a day [26]. Some hoverfly species migrate across great distances, even across seas [16], [44], [45].

Percent cover of each of the 15 land use types was used to calculate landscape diversity (*H*) on each of the five spatial scales, using the Shannon index [46]. Only two out of the thirteen land-use types (unimproved grassland and improved grassland) were present at all sites and in all landscape sectors. However, the two were collinear (variance inflation factors (VIF) above 3 [47]). Unimproved grassland was the dominant land-use type in the study landscapes and was negatively correlated with landscape diversity (5****km radius: r = −0.6893, P = 0.001), and so unimproved grassland was used as an inverse indicator of landscape complexity.

Linear landscape features contribute to the complexity of a landscape and thus impact on biodiversity. In our study landscape, hedgerows are the dominant linear feature and consequently may affect plant richness. We estimated the percentage cover and length (km) of hedgerows and the number of connections between hedgerows within a 1****km radius around each site (larger scales could not be included due to budgetary restrictions). These three hedgerow measures were not found to be collinear (variance inflation factors (VIF) below 3 [47]). The hedgerow measures were also not significantly related to landscape complexity (i.e. percentage unimproved grassland) within the 1****km scale (unpublished data). This is likely to be due to differences between data sources used: the CORINE database (used to calculate landscape complexity) has a low resolution that does not include linear habitats smaller than 100 m^2^ whereas visual inspection of aerial photographs (used to calculate hedgerow measures) can pinpoint smaller linear features and their connections. These hedgerow measures may give an indication of hedgerow structure within the landscape which is known to impact on plant diversity [48], [49].

The isolation of sites, i.e. the distance (in metres) from each study site to the nearest edges of 10 habitat types, was calculated in relation to: broadleaved forest, coniferous forest, mixed forest, transitional woodland scrub, urban areas, bogs, marshes, heaths, stream courses and water bodies. Most of the 10 habitat types were found to be collinear (variance inflation factors (VIF) above 3 [47]) so were pooled into four categories: forest, urban, wetland and open water. For example: broadleaved forest, coniferous forest, mixed forest and transitional woodland scrub were pooled into the category named forest. All landscape analyses were done using ArcGIS 9.3.1, ESRI, Redlands, CA, USA.

### Statistical Analysis

Plant community similarity (in terms of percentage cover of each species) between organic and conventional field edges and centres was graphically analysed using Non-Metric Multidimensional Scaling (NMDS) and significant differences were tested for using PERMANOVA+ (fixed factors were transect position (edge/centre), farm type (organic/conventional) and their interaction while random factors were field (1–3) and farm pair (1–10)), using PRIMER 6.1.13 [37]. To determine which plant species were primarily providing the discrimination (i.e. highest percentage contribution to the plant community) between organic and conventional field edges and centres, we performed SIMPER analysis using PRIMER 6.1.13 [37].

Plant richness was calculated for all plants (“total plants”), for insect-pollinated forbs, non-insect pollinated forbs and graminoids as the cumulative species richness of each transect. Each measure of richness was analysed in relation to: (1) local factors (farming system, position in the field and soil parameters (PC1, PC2 and PC4)) and landscape complexity for each of the five spatial scales; (2) local factors (farming system and position in the field) and linear landscape features and (3) local factors (farming system and position in the field) and distance metrics using Linear Mixed Effects Models. As individual soil parameters (K, P, Mg and pH) were collinear and so could not be analysed in the same model without using PCA scores (see Soil Analysis section), each parameter was also analysed using Linear Mixed Effects Models to elucidate individual soil parameter differences between farming system and position in the field. Landscape complexity and linear feature data were analysed in separate models because landscape complexity data was available for each of 1–5****km scales but only data for the 1****km scale was available for linear features. We accounted for the hierarchical structure of the data by including random terms: farm pair (1–10), farm (1–20) and field (1–3). In each local and landscape model, the fixed effects included: farm type (organic/conventional), location-within-field (edge/centre), soil parameters (PC1, PC2, PC4) and percent cover of unimproved grassland. In the linear landscape features models, summed hedgerow length, hedgerow percent cover, number of hedgerow connections, farm type and location-within-field were included as fixed effects. In the distance metrics models, farm type, location-within-field, distances to forest, urban, wetland and open water were included as fixed effects. In the individual soil parameter model, the fixed effects included: farm type and location-within-field. Biologically relevant two-way and three-way interactions between fixed effects were included. Models were simplified by removing, first, non-significant interactions (*P*>0.05) and then any non-significant main effects (that were not constituent within a significant interaction). Models were validated by: plotting standardised residuals against fitted values and each explanatory variable to assess homogeneity and independence, verifying normality of residuals using Normal QQ-plots and assessing the models for influential observations using Cook distances [50]. Mixed modelling was carried out using the nlme [51] package in *R* [52].

## Results

A total of 69 plant species were found, with 61 in organic and 41 in conventional farms (Table S2). There were 31 insect-pollinated forbs (26 in organic, 20 in conventional), 18 non-insect pollinated forbs (17 in organic, 14 in conventional) and 20 graminoids (18 in organic, 14 in conventional). Of the non-insect pollinated species, 12 were normally self-pollinated and 6 were wind pollinated.

### Plant Community Composition

**Figure 2:**
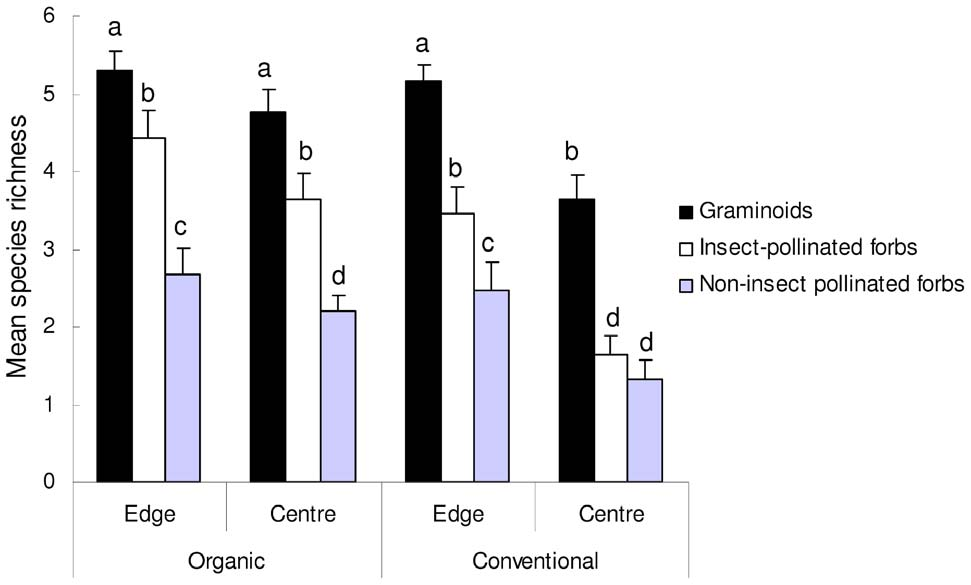
Mean (± standard error) graminoid/forb richness per transect in organic/conventional field edges and centres. Letters above the bars indicate significant differences among plant groups, farm type and edge/centre, using Linear Mixed Effects Models.

**Figure 3:**
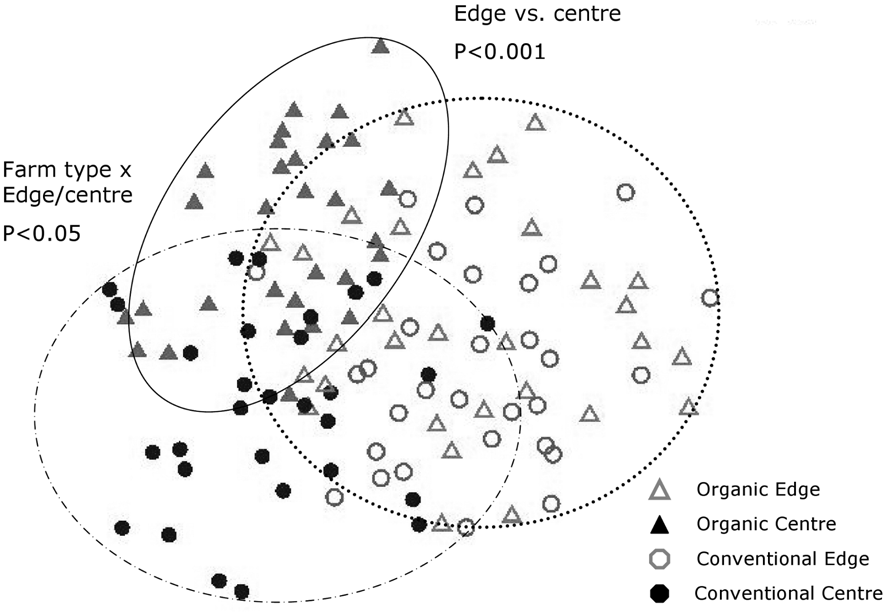
Plant community similarity between organic and conventional edge and centre transects. Illustrated using Non-metric Multidimensional Scaling (MNS with Bray - Curtis index, 2 Axes, 2D Stress  = 0.21), with significance values obtained using PERMANOVA+. Three distinct plant community groups emerged: organic and conventional edges (dotted line); organic centres (continuous line) and conventional centres (dashed and dotted line).

Graminoids dominated the community in organic and conventional field edges and centres, followed by insect-pollinated forbs (Fig. 2). Non-insect pollinated forbs were far less frequent on all farms (Fig. 2). Multivariate analysis showed significant differences in plant community composition between organic and conventional farms (Pseudo-F = 4.895, P = 0.009) with conventional farm plant communities representing a subset of those in organic farms. There was a significant interaction between farm type and edge/centre (Pseudo-F = 1.320, P = 0.042) (Fig. 3) with the centres of conventional fields mainly characterised by graminoids (89% contribution to community (in terms of cover and frequency), particularly *Lolium perenne* and *Agrostis stolonifera*) while organic centres were characterised by a large percentage of insect-pollinated forbs (42% contribution to community, *Trifolium repens* and *Ranunculus repens*) and much fewer non-insect-pollinated forbs (2% contribution to community, *Taraxacum* spp.) as well as graminoids (46% contribution to community, *Lolium perenne, Holcus lanatus* and *Agrostis spp.*). Community composition in organic and conventional field edges was similar but all edges were significantly different from all field centres (Pseudo-F = 20.899, P = 0.001) (Fig. 3) as they contained varying mixtures of insect-pollinated forbs (10–14% contribution to community), non-insect pollinated forbs (6–7% contribution to community) and graminoids (70–75% contribution to community).

### Plant Richness: Local and Landscape Factors

Total plant richness, and that of insect-pollinated forbs and graminoids, were related to local factors and landscape factors, while non-insect pollinated forb richness was related to local factors only (Table 2). Total plant richness was significantly higher on organic farms than conventional and also higher in field edges than centres. Conventional field centres were particularly depauperate in terms of species richness (Fig. 2). Total plant richness was also significantly positively related to the PC1 scores of the principal components analysis (K soil parameter) but unrelated to PC2 or PC4 (see Table S3 for more detailed soil analysis results). The richness of insect-pollinated forbs and graminoids followed the same patterns as total plant richness in terms of local site characteristics but non-insect pollinated forbs did not. Non-insect pollinated forb richness was similar in both farming systems but was higher in all field edges compared to all field centres (Fig. 2). Non-insect pollinated forbs were also significantly positively related to PC1 scores only.

**Table 2:** Interacting relationships among local and landscape factors and plant richness (total plants, insect-pollinated forbs, non-insect pollinated forbs and graminoids) at five different spatial scales (1–5****km radii around sites).

		Local												Landscape		Local x landscape					
	Scale	FT		E/C		FT x E/C		PC1		PC2		PC4		%UG		FT x %UG		E/C x %UG		FT x E/C x %UG	
Richness	km	t_d.f._	P	t_d.f._	P	t_d.f._	P	t_d.f._	P	t_d.f._	P	t_d.f._	P	t_d.f._	P	t_d.f._	P	t_d.f._	P	t_d.f._	P
Total plants	1	3.092_8_	0.015	5.612_56_	0.000	−3.007_56_	0.004	4.032_56_	0.000	−	N.S.	–	N.S.	–	N.S.	–	N.S.	−2.537_56_	0.014	–	N.S.
	2	2.964_9_	0.016	7.290_57_	0.000	−2.485_57_	0.016	4.192_57_	0.000	–	N.S.	–	N.S.	–	N.S.	–	N.S.	–	N.S.	–	N.S.
	3	3.005_8_	0.017	4.903_56_	0.000	−2.795_56_	0.007	2.267_56_	0.000	–	N.S.	–	N.S.	–	N.S.	–	N.S.	−2.227_56_	0.030	–	N.S.
	4	2.960_8_	0.018	5.251_56_	0.000	−2.634_56_	0.011	4.228_56_	0.000	–	N.S.	–	N.S.	–	N.S.	–	N.S.	−2.674_56_	0.010	–	N.S.
	5	2.918_8_	0.019	5.255_56_	0.000	−2.528_56_	0.014	4.207_56_	0.000	–	N.S.	–	N.S.	–	N.S.	–	N.S.	−2.805_56_	0.007	–	N.S.
Insect-pollinated forbs	1	4.314_8_	0.003	4.895_56_	0.000	−2.647_56_	0.010	2.455_56_	0.017	–	N.S.	–	N.S.	–	N.S.	–	N.S.	−2.632_56_	0.011	–	N.S.
	2	4.075_9_	0.003	5.557_57_	0.000	−2.102_57_	0.040	2.728_57_	0.008	–	N.S.	–	N.S.	–	N.S.	–	N.S.	–	N.S.	–	N.S.
	3	4.284_8_	0.003	4.044_56_	0.000	−2.379_56_	0.021	2.697_56_	0.009	–	N.S.	–	N.S.	–	N.S.	–	N.S.	−2.050_56_	0.045	–	N.S.
	4	4.186_8_	0.003	4.273_56_	0.000	−2.214_56_	0.031	2.661_56_	0.010	–	N.S.	–	N.S.	–	N.S.	–	N.S.	−2.356_56_	0.022	–	N.S.
	5	4.137_8_	0.003	4.179_56_	0.000	−2.115_56_	0.039	2.630_56_	0.011	–	N.S.	–	N.S.	–	N.S.	–	N.S.	−2.115_56_	0.039	–	N.S.
Non-insect pollinated forbs	1	–	N.S.	3.996_58_	0.000	–	N.S.	2.741_58_	0.008	–	N.S.	–	N.S.	–	N.S.	–	N.S.	–	N.S.	–	N.S.
	2	–	N.S.	3.996_58_	0.000	–	N.S.	2.741_58_	0.008	–	N.S.	–	N.S.	–	N.S.	–	N.S.	–	N.S.	–	N.S.
	3	–	N.S.	3.996_58_	0.000	–	N.S.	2.741_58_	0.008	–	N.S.	–	N.S.	–	N.S.	–	N.S.	–	N.S.	–	N.S.
	4	–	N.S.	3.996_58_	0.000	–	N.S.	2.741_58_	0.008	−	N.S.	−	N.S.	–	N.S.	–	N.S.	–	N.S.	–	N.S.
	5	–	N.S.	3.996_58_	0.000	–	N.S.	2.741_58_	0.008	–	N.S.	–	N.S.	–	N.S.	–	N.S.	–	N.S.	–	N.S.
Graminoids	1	2.944_8_	0.017	5.833_53_	0.000	−3.560_53_	0.001	3.874_53_	0.000	–	N.S.	–	N.S.	–	N.S.	–	N.S.	−3.089_53_	0.003	–	N.S.
	2	2.891_8_	0.020	4.892_53_	0.000	−3.473_53_	0.001	4.007_53_	0.000	–	N.S.	–	N.S.	–	N.S.	–	N.S.	−2.265_53_	0.028	–	N.S.
	3	2.732_9_	0.023	6.420_54_	0.000	−3.067_54_	0.003	3.801_54_	0.000	–	N.S.	–	N.S.	–	N.S.	–	N.S.	–	N.S.	–	N.S.
	4	2.732_9_	0.023	6.420_54_	0.000	−3.067_54_	0.003	3.801_54_	0.000	–	N.S.	–	N.S.	–	N.S.	–	N.S.	–	N.S.	–	N.S.
	5	2.732_9_	0.023	6.420_54_	0.000	−3.067_54_	0.003	3.801_54_	0.000	–	N.S.	–	N.S.	–	N.S.	–	N.S.	–	N.S.	–	N.S.

Analysed using Linear Mixed Effects Models. Note: FT  =  Farm type (organic or conventional); E/C  =  Edge/Centre (position in the field); %UG  =  Percentage unimproved grassland (which reflects an inverse measure of landscape complexity); PC1 and PC2 =  first and second principal components from a principal component analysis on soil parameters; N.S.  =  not significant.

The plant categories varied in terms of their relationships with landscape structure at different spatial scales (Table 2). Total plant and insect-pollinated forb richness in the field edges was significantly negatively related to the percent cover of unimproved grassland in the landscape between the scales of 1 and 5****km (though with no relationship evident at the 2****km scale). In contrast, graminoid richness in the field edges was negatively related to the percent cover of unimproved grassland in the 1****km and 2****km scales only, while non-insect pollinated forbs were unrelated to landscape structure at any spatial scale. The richness of all plants, insect-pollinated forbs, non-insect pollinated forbs and graminoids in all field centres were not related to the percent cover of unimproved grassland at any spatial scale. No interaction effect was found between farm type and the percent cover of unimproved grassland in any spatial scale on plant richness in general.

#### Linear features

Hedgerows were the dominant linear landscape feature around sites and covered a substantial percentage of the landscape within 1****km radii (Table S4). There were on average 5–6****km of hedgerows and approximately 30 hedgerow connections within these 1****km radii (with more hedgerow connections surrounding conventional farms than organic). Plant richness was significantly related to the various hedgerow measures. Total plant richness and insect-pollinated forb richness in organic field edges increased with increasing hedgerow length and area respectively, i.e. total plant richness was positively related to the interaction between farm type, edge/centre and hedgerow length while insect-pollinated forb richness was positively related to the interaction between farm type, edge/centre and hedgerow area (Table 3). Non-insect pollinated forb richness was: positively related to hedgerow area; negatively related to the interaction between hedgerow area and number of hedgerow connections; and positively related to the interaction between hedgerow length and number of connections. Graminoid richness in the edges of organic and conventional fields was positively related to hedgerow area.

**Table 3:** Relationships between plant richness (total plants, insect-pollinated forbs, non-insect pollinated forbs and graminoids) and hedgerow area, hedgerow length and number of hedgerow connections (and various interactions between factors) within 1****km radii around sites.

	HA		HL		Con		FT x E/C x HA		FT x E/C x HL		E/C x HA		HA x Con		HL x Con	
Richness	t_d.f._	P	t_d.f._	P	t_d.f._	P	t_d.f._	P	t_d.f._	P	t_d.f._	P	t_d.f._	P	t_d.f._	P
Total plants	–	N.S.	–	N.S.	–	N.S.	–	N.S.	2.242_56_	0.029	–	N.S.	–	N.S.	–	N.S.
Insect-pollinated forbs	–	N.S.	–	N.S.	–	N.S.	2.603_56_	0.012	–	N.S.	–	N.S.	–	N.S.	–	N.S.
Non-insect pollinated forbs	3.57_5_	0.016	–	N.S.	–	N.S.	–	N.S.	–	N.S.	–	N.S.	−3.041_5_	0.029	2.648_5_	0.046
Graminoids	–	N.S.	–	N.S.	–	N.S.	–	N.S.	–	N.S.	2.039_57_	0.046	–	N.S.	–	N.S.

Explanatory variables that were not significantly related with any response variable are not included in this table. Analysed using Linear Mixed Effects Models. Note: HA  =  Hedge area (%); HL  =  Hedge length (km); Con  =  number of connections between hedges; FT  =  Farm type (organic or conventional); E/C  =  Edge/centre (position in the field); N.S.  =  not significant.

#### Distance metrics

Insect-pollinated forb richness significantly increased with increasing proximity to wetlands (t_d.f._  = −2.405_8_, *P* = 0.043). There was no relationship between insect-pollinated plant richness and proximity to woodland, urban areas or open water and no interaction effects between farm type, edge/centre and distance metrics on this plant group. Total plant, non-insect-pollinated forbs and graminoid richness were not related to distance metrics.

## Discussion

### Local Factor Influences on Plant Communities

Total plant richness was higher in organic dairy farms compared to conventional farms because there was a higher plant richness in organic field centres than conventional centres; no difference was found between organic and conventional field edges. Similar patterns were evident in mixed arable and grassland systems in the UK [23] but not in Danish dairy systems [22].

It is not clear, which organic dairy farm management activities (lower fertilisation levels, lower stocking rates or lack of chemical herbicide applications) benefit plant diversity most or in which combination. In our study, macronutrients did not seem to be the most significant driver of plant diversity differences between organic and conventional systems. Though the macronutrient potassium was significantly related to plant richness, there was no interaction effect between any soil factor and farm type on plant richness and there were no significant differences among potassium levels in organic and conventional field edges and centres (Table S3). We did not include all macro-nutrients (e.g. nitrogen) in our soil analyses and this may lead to an underestimation of the influence of soil parameters on plant species richness in our study, but nitrogen enrichment of the soil (compared to other macronutrients such as phosphorus) has not been found to be the most important driver of species loss in semi-natural grasslands [53]. Though nutrient enrichment of the soil [53], can be responsible for species loss from semi-natural grasslands, in species-poor habitats, such as the commercially productive organic and conventional grasslands in our study, the effects of nutrient enrichment may become less important. This may be because most of the plant species that remain are likely to be relatively tolerant of high nutrient conditions. Previous studies in organic arable systems suggest that the exclusion of chemical herbicides in organic farming is important in explaining increased plant species richness in field centres [see: 24]. It seems likely that this is also the case in organic dairy field centres – chemical herbicides were applied annually in all the conventional farms in our study. However intensive grazing can also be responsible for species loss from semi-natural grasslands [54]. The conventional farms in our study had, on average, higher stocking densities. Therefore, more experimental research into the effects of organic and conventional farm management activities on species diversity is needed to elucidate the drivers of plant community composition in intensive dairy systems.

Unlike the finding for arable systems [5], there were more insect-pollinated forbs than non-insect pollinated forbs in both organic and conventional grasslands. Thus, intensive grassland plant communities appear to differ fundamentally from those of arable systems in terms of their relationships with pollinators and their plant-pollinator interaction networks, mutual dependence between plant and pollinator communities being more important in grassland than arable systems. If this is indeed the case, then changes in pollinator communities within grassland systems may have greater ramifications for plant-pollinator network stability than similar changes in arable systems.

Within the grassland systems in our study, plant community composition varied with farming system and position in the field. Considerably more forbs were present in organic field centres than conventional field centres. The forbs present in organic field centres were mainly insect-pollinated e.g. *Trifolium repens* and *Ranunculus repens*. While *T. repens* is a common component in organic seed mixtures [55], other insect-pollinated forbs are not and their increased presence in organic field centres could be beneficial to pollinators, attracting more insects and thus improving pollination services [12] (of course, certain forbs are potentially harmful to livestock, as is the case with *R. repens* and other buttercups [56], [57]).

### Local Versus Landscape Factor Influences on Plant Richness

Our results indicate that farm management activities influenced insect-pollinated forb richness in field centres, while increasing landscape complexity (calibrated by decreasing percentage of unimproved grassland within 1, 3, 4 and 5****km radii around sites) positively influenced insect-pollinated forb richness in field edges. Field edges tend to be less affected than field centres by farm management activities and are thus closer to a semi-natural condition [49]. Wild bees are important pollinators of wild plants [1] and they too can be influenced by landscape structure at scales of up to 3****km [41]. Thus, insect-pollinated forb richness at the field scale may be influenced by landscape complexity at large scales, mirroring the situation with some of the pollinators on which they depend. Parallel patterns have been recorded with insect-pollinated plant versus pollinator diversity in Great Britain and the Netherlands [3].

It is not clear why landscape complexity within the 2****km scale had no effect on insect-pollinated forb richness in field edges but may reflect the fact that relationships between local diversity, local factors and landscape structure change depending on the scale one is focusing on [58]. This illustrates the importance of choosing the correct scale in order to manage plants and pollinators in the landscape and, indeed, our study indicates that some scales may be more important for some plant groups than for others.

In contrast to the findings for insect-pollinated forbs, non-insect pollinated forb richness was unrelated to landscape complexity at any spatial scale – it was only related to position in the field and soil parameters. This may be because the majority of non-insect pollinated forbs in our study were self-pollinated. Graminoids in field edges were positively related to landscape complexity at lower scales than insect-pollinated forbs (as well as to farm type, position in the field and soil parameters). The reasons for this relationship between field edge graminoids and the landscape are unclear, but may be related to the fact that the graminoids in our study are wind pollinated [35] and so pollen transfer within the landscape could be dependent on climatic variables and landscape structure at smaller scales than for the factors important for insect-pollinated forbs. Our findings show the importance of using information on the pollination system of plants in order to understand their relationships with landscape structure. This is further illustrated by our findings for ‘total plants’ vs. landscape complexity which mirror the findings for insect-pollinated plants (the most species-rich group over all sites) but contrast greatly with findings for non-insect pollinated plants. Most landscape studies focus on total plant richness but our findings show that this may obscure the highly variable impacts of landscape structure on plant groups with different pollination systems.

Plant richness was also strongly related to linear features (hedgerows) within 1****km around sites. There was variation between plant groups in terms of which hedgerow measure was important, but hedgerow area, length or the interaction between these and the number of hedgerow connections were all significantly positively correlated with plant richness. Hedgerow structure is known to play an important role in determining hedgerow plant species distributions [48] and our results indicate that different plant groups (based on mode of pollination and ecology, i.e. graminoid vs. forb) respond differently to different aspects of hedgerow structure. There are many possible reasons for this. Hedgerows are known to act as corridors for movement of many plant species [49] and can act as barriers to or facilitators of pollen flow. Pollinators interact with linear landscape features [26], [27], [59] and hedgerows have been found to facilitate pollen dispersal between insect-pollinated forb populations through pollinator movements [60]. Hedgerows can also interrupt or slow down air fluxes [61] thus possibly affecting pollen dispersal in wind pollinated species. Maintenance or restoration of hedgerow networks in the landscape favours biodiversity but little is known of how hedgerow structure, pollen dispersal and plant community composition interact in the landscape.

Habitat fragmentation is known to affect different plant species in different ways [31] and so we tested whether the richness of insect-pollinated plants, non-insect pollinated plants and graminoids were related to proximity to different fragmented habitats, such as woodlands and wetlands. We found that insect-pollinated forb richness increased with proximity to wetlands only but found no relation between distance metrics and non-insect pollinated forbs or graminoids. The proximity of wetlands may boost plant richness as they will provide habitat for a range of species and may form a source from which colonisation can take place. The reasons for specific benefits to insect-pollinated plant richness are unclear, but may derive from a benefit to pollinator species from the proximity of wetlands: many pollinators (including hoverfly and solitary bee species) utilise wetlands as larval and forage habitats [16], [30].

### Conclusion

Plant community composition within intensive dairy grasslands, though species poor, differs from arable plant communities in having much higher proportions of insect-pollinated forbs in the community. This is likely to have implications for insect pollinator communities and plant-pollinator interaction networks. Organic farming was found to support greater plant richness in field centres than did conventional farming. The plant communities within field edges (organic or conventional) exhibit relationships with landscape structure that varied depending on their principal pollen vector. Our results indicate that insect-pollinated forbs in field edges differ from non-insect pollinated plant groups in terms of the extent to which landscape complexity at large spatial scales determines local richness. Such a relationship has not been demonstrated previously but comparisons can be drawn from the literature between this relationship and that found for pollinators, particularly bees, and landscape structure [41]. Our study also indicates that linear landscape features (hedgerows) and proximity to certain habitats influence the species richness of plant groups in grasslands in different ways, depending on their pollination system. The relationships between insect-pollinated forbs, their pollinators and landscape structure at different spatial scales need to be explored further, as all are inextricably linked.

## Supporting Information

Figure S1 **Percentage cover of unimproved grassland around each study site (farm pairs 1–10) for each of five spatial scales (1–5 km radii around sites).**
(DOC)

Table S1 **Mean ± standard error soil parameters measured in organic and conventional field edges and centres.**
(DOC)

Table S2 **List of all insect-pollinated forbs, non-insect pollinated forbs and graminoids and associated mean percentage cover per (0.5×0.5 m) quadrat (n = 600) recorded in the edges and centres of organic and conventional dairy fields.**
(DOC)

Table S3 **Relationships between soil parameters and farm type (organic/conventional), position in the field (edge/centre) and the interaction between farm type and edge/centre.**
(DOC)

Table S4 **Mean ± standard error hedgerow area, hedgerow length and number of connections between hedgerows within 1 km radii of organic and conventional farms.**
(DOC)

## References

[1] Buchmann SL, Nabhan GP (1996). The Forgotten Pollinators..

[2] Steffan-Dewenter I, Potts SG, Packer L (2005). Pollinator diversity and crop pollination services are at risk.. Trends in Ecology & Evolution.

[3] Biesmeijer JC, Roberts SPM, Reemer M, Ohlemuller R, Edwards M (2006). Parallel declines in pollinators and insect-pollinated plants in Britain and the Netherlands.. Science.

[4] Baker HG (1974). The Evolution of Weeds.. Annual Review of Ecology and Systematics.

[5] Gabriel D, Tscharntke T (2007). Insect pollinated plants benefit from organic farming.. Agriculture, Ecosystems & Environment.

[6] Regal PJ (1982). Pollination by Wind and Animals: Ecology of Geographic Patterns.. Annual Review of Ecology and Systematics.

[7] Plantureux S, Peeters A, McCracken D (2005). Biodiversity in intensive grasslands: Effect of management, improvement and challenges.. Agronomy Research.

[8] Vickery JA, Tallowin JR, Feber RE, Asteraki EJ, Atkinson PW (2001). The management of lowland neutral grasslands in Britain: effects of agricultural practices on birds and their food resources.. Journal of Applied Ecology.

[9] Department of Agriculture (2009). Fact sheet on Irish Agriculture.

[10] McMahon BJ, Helden A, Anderson A, Sheridan H, Kinsella A (2010). Interactions between livestock systems and biodiversity in South-East Ireland.. Agriculture, Ecosystems & Environment.

[11] Santorum V, Breen J (2005). Bumblebee diversity on Irish farmland.. Irish journal of agri-environmental research.

[12] Power EF, Stout JC (2011). Organic dairy farming: impacts on insect–flower interaction networks and pollination.. Journal of Applied Ecology.

[13] Martin JR, Gabbett M, Perrin PM, Delaney A (2007). Semi-natural grassland survey of counties Roscommon and Offaly..

[14] Ivimey-Cook RB, Proctor MCF (1966). Plant Communities of the Burren, Co. Clare.. Proceedings of The Royal Irish Academy Dublin.

[15] Fitzpatrick Ú, Murray TE, Paxton RJ, Breen J, Cotton D (2007). Rarity and decline in bumblebees - A test of causes and correlates in the Irish fauna.. Biological Conservation.

[16] Speight MCD (2008). Database of Irish Syrphidae (Diptera)..

[17] Kleijn D, Verbeek M (2000). Factors affecting the species composition of arable field boundary vegetation.. Journal of Applied Ecology.

[18] Schippers P, Joenje W (2002). Modelling the effect of fertiliser, mowing, disturbance and width on the biodiversity of plant communities of field boundaries.. Agriculture, Ecosystems & Environment.

[19] Bengtsson J, Ahnstrom J, Weibull AC (2005). The effects of organic agriculture on biodiversity and abundance: a meta-analysis.. Journal of Applied Ecology.

[20] Morandin LA, Winston ML (2005). Wild bee abundance and seed production in conventional, organic, and genetically modified canola.. Ecological Applications.

[21] Romero A, Chamorro L, Sans FX (2008). Weed diversity in crop edges and inner fields of organic and conventional dryland winter cereal crops in NE Spain.. Agriculture, Ecosystems & Environment.

[22] Petersen S, Axelsen JA, Tybirk K, Aude E, Vestergaard P (2006). Effects of organic farming on field boundary vegetation in Denmark.. Agriculture, Ecosystems & Environment.

[23] Gabriel D, Sait SM, Hodgson JA, Schmutz U, Kunin WE (2010). Scale matters: the impact of organic farming on biodiversity at different spatial scales.. Ecology Letters.

[24] Rundlöf M, Edlund M, Smith HG (2010). Organic farming at local and landscape scales benefits plant diversity.. Ecography.

[25] Roschewitz I, Gabriel D, Tscharntke T, Thies C (2005). The effects of landscape complexity on arable weed species diversity in organic and conventional farming.. Journal of Applied Ecology.

[26] Wratten SD, Bowie MH, Hickman JM, Alison ME, Sedcole JR (2003). Field Boundaries as Barriers to Movement of Hover Flies (Diptera: Syrphidae) in Cultivated Land.. Oecologia.

[27] Cranmer L, McCollin D, Ollerton J (2012). Landscape structure influences pollinator movements and directly affects plant reproductive success.. Oikos.

[28] Jacobs JH, Clark SJ, Denholm I, Goulson D, Stoate C (2009). Pollination biology of fruit-bearing hedgerow plants and the role of flower-visiting insects in fruit-set.. Annals of Botany.

[29] Ouin A, Burel F (2002). Influence of herbaceous elements on butterfly diversity in hedgerow agricultural landscapes.. Agriculture Ecosystems & Environment,.

[30] Michener CD, Baltimore (2007). The Bees of the World.. Md.

[31] Dauber J, Biesmeijer JC, Gabriel D, Kunin WE, Lamborn E (2010). Effects of patch size and density on flower visitation and seed set of wild plants: a pan-European approach.. Journal of Ecology.

[32] Stace CA (2010). New flora of the British Isles..

[33] Grime JP, Hodgson JG, Hunt R (2007). Comparative Plant Ecology: A Functional Approach to Common British Species: Castlepoint Press, London.

[34] Klotz S, Kühn I, Durka W (2002). BIOLFLOR - Eine Datenbank zu biologisch-ökologischen Merkmalen der Gefäßpflanzen in Deutschland..

[35] Cope T, Gray A (2009). Grasses of the British Isles BSBI Handbook no 13..

[36] Alexander S, Black A, Boland A, Burke J, Carton OT, Coulter BS, Lalor S (2008). Major and micro nutient advice for productive agricultural crops.. 50th anniversary edition ed: Teagasc, Johnstown Castle, Co Wexford.

[37] Clarke KR, Gorley RN (2006). PRIMER v6: User Manual/Tutorial..

[38] Bossard M, Feranec J, Otahel J (2000). CORINE land cover technical guide - Addendum 2000..

[39] European Environment Agency (2007). CLC2006 Technical guidelines..

[40] Sullivan CA, Skeffington MS, Gormally MJ, Finn JA (2010). The ecological status of grasslands on lowland farmlands in western Ireland and implications for grassland classification and nature value assessment.. Biological Conservation.

[41] Steffan-Dewenter I, Munzenberg U, Burger C, Thies C, Tscharntke T (2002). Scale-dependent effects of landscape context on three pollinator guilds.. Ecology.

[42] Westphal C, Steffan-Dewenter I, Tscharntke T (2006). Bumblebees experience landscapes at different spatial scales: possible implications for coexistence.. Oecologia.

[43] Haenke S, Scheid B, Schaefer M, Tscharntke T, Thies C (2009). Increasing syrphid fly diversity and density in sown flower strips within simple vs. complex landscapes.. Journal of Applied Ecology.

[44] Conn DLT (1976). Estimates of Population Size and Longevity of Adult Narcissus Bulb Fly Merodon equestris Fab. (Diptera: syrphidae).. Journal of Applied Ecology.

[45] Dziock F (2006). Life-History Data in Bioindication Procedures, Using the Example of Hoverflies (Diptera, Syrphidae) in the Elbe Floodplain.. International Review of Hydrobiology.

[46] Krebs JR, Wilson JD, Bradbury RB, Siriwardena GM (1999). The second silent spring?. Nature.

[47] Zuur A, Ieno EN, Smith GM (2007). Analysing Ecological Data..

[48] Deckers B, Hermy M, Muys B (2004). Factors affecting plant species composition of hedgerows: relative importance and hierarchy.. Acta Oecologica.

[49] Le Coeur D, Baudry J, Burel F, Thenail C (2002). Why and how we should study field boundary biodiversity in an agrarian landscape context.. Agriculture, Ecosystems & Environment.

[50] Zuur AF, Ieno EN, Walker NJ, Saveliev AA, Smith GM (2009). Mixed Effects Models and Extensions in Ecology with R New York: Springer.

[51] Pinheiro J, Bates D, DebRoy S, Sarkar D, the R Core team (2009). nlme: Linear and Nonlinear Mixed Effects Models. R package version 3.1-96.. http://CRAN.R-project.org/package=nlme.

[52] R Development Core Team (2007). R: A language and environment for statistical computing. R Foundation for Statistical Computing Vienna, Austria. ISBN 3-900051-07-0.. http://www.R-project.org.

[53] Ceulemans T, Merckx R, Hens M, Honnay O (2011). A trait-based analysis of the role of phosphorus vs. nitrogen enrichment in plant species loss across North-west European grasslands.. Journal of Applied Ecology.

[54] Olff H, Ritchie ME (1998). Effects of herbivores on grassland plant diversity.. Trends in Ecology & Evolution.

[55] Irish Organic Farmers, Growers Association (2006). The IOFGA Standards For Organic Food and Farming in Ireland - incorporating amendments A1 to.

[56] James LF, Keeler RF, Bailey EMJ, Cheeke PR, Hegarty MP.PoisonousPlants; (1992). Iowa State University Press, Ames.

[57] Parton K, Bruere AN (2002). Plant poisoning of livestock in New Zealand.. New Zealand Veterinary Journal.

[58] Dauber J, Purtauf T, Allspach A, Frisch J, Voigtländer K (2005). Local vs. landscape controls on diversity: a test using surface-dwelling soil macroinvertebrates of differing mobility.. Global Ecology & Biogeography.

[59] Ekroos J, Piha M, Tiainen J (2008). Role of organic and conventional field boundaries on boreal bumblebees and butterflies.. Agriculture, Ecosystems & Environment.

[60] Van Geert A, Van Rossum F, Triest L (2010). Do linear landscape elements in farmland act as biological corridors for pollen dispersal?. Journal of Ecology.

[61] Burel F (1996). Hedgerows and their role in agricultural landscapes.. Critical Reviews in Plant Sciences.

