# Supplementary material for: Organic Farming and Landscape Structure: Effects on Insect-Pollinated Plant Diversity in Intensively Managed Grasslands
Source: PLoS One. 2012 May 30;7(5):e38073. doi: 10.1371/journal.pone.0038073 (PMC3364189; doi:10.1371/journal.pone.0038073)
Supplement: Figure S1 — Percentage cover of unimproved grassland around each study site (farm pairs 1–10) for each of five spatial scales (1–5 km radii around sites). (DOC) [file pone.0038073.s001.doc]

Figure S1: The percentage cover of unimproved grassland around each study site (farm pairs 1-10) for each of five spatial scales (1-5km radii around sites).
