# Supplementary material for: Organic Farming and Landscape Structure: Effects on Insect-Pollinated Plant Diversity in Intensively Managed Grasslands
Source: PLoS One. 2012 May 30;7(5):e38073. doi: 10.1371/journal.pone.0038073 (PMC3364189; doi:10.1371/journal.pone.0038073)
Supplement: Table S1 — Mean ± standard error soil parameters measured in organic and conventional field edges and centres. (DOC) [file pone.0038073.s002.doc]

Table S1: Mean ± standard error soil parameters measured in organic and conventional field edges and centres.

|  | Organic | | Conventional | |
| --- | --- | --- | --- | --- |
|  | Edge | Centre | Edge | Centre |
| Soil parameter | Mean ± SE | Mean ± SE | Mean ± SE | Mean ± SE |
| Phosphorus (mg/l) | 21.183 ± 4.661 | 5.283 ± 0.700 | 13.223 ± 2.107 | 10.587 ± 2.084 |
| Potassium (mg/l) | 201.907 ± 20.038 | 118.093 ± 10.445 | 209.827 ± 21.586 | 141.977 ± 16.740 |
| Magnesium (mg/l) | 245.293 ± 18.410 | 159.93 ± 9.151 | 267.813 ± 22.289 | 273.713 ± 28.534 |
| pH | 5.778 ± 0.133 | 5.486 ± 0.071 | 5.799 ± 0.128 | 5.559 ± 0.070 |
