# Supplementary material for: Organic Farming and Landscape Structure: Effects on Insect-Pollinated Plant Diversity in Intensively Managed Grasslands
Source: PLoS One. 2012 May 30;7(5):e38073. doi: 10.1371/journal.pone.0038073 (PMC3364189; doi:10.1371/journal.pone.0038073)
Supplement: Table S2 — List of all insect-pollinated forbs, non-insect pollinated forbs and graminoids and associated mean percentage cover per (0.5×0.5 m) quadrat (n = 600) recorded in the edges and centres of organic and conventional dairy fields. (DOC) [file pone.0038073.s003.doc]

Table S2: List of all insect-pollinated forbs, non-insect pollinated forbs and graminoids and associated mean percentage cover per (0.5x0.5m) quadrat (n = 600) recorded in the edges and centres of organic and conventional dairy fields.

| **Species groups (mean % cover)** | **Organic** | | **Conventional** | |
| --- | --- | --- | --- | --- |
| **Insect-pollinated forbs** | **Edge** | **Centre** | **Edge** | **Centre** |
| *Achillea millefolium* | 0.07 | 0.07 | 0 | 0.10 |
| *Cardamine pratensis* | 0.15 | 0.34 | 0.03 | 0.22 |
| *Cirsium arvensis* | 0.61 | 0.41 | 1.74 | 0.26 |
| *Cirsium vulgaris* | 0.87 | 1.20 | 0.91 | 0.05 |
| *Heracleum sphondylium* | 0.25 | 0 | 0.23 | 0 |
| *Potentilla anserina* | 0.37 | 0.03 | 0 | 0 |
| *Prunella vulgaris* | 0.03 | 0 | 0.03 | 0 |
| *Ranunculus acris* | 0.02 | 0.21 | 0 | 0 |
| *Ranunculus bulbosus* | 0 | 0.01 | 0 | 0 |
| *Ranunculus repens* | 2.93 | 3.22 | 3.23 | 1.10 |
| *Senecio jacobaea* | 0.30 | 0.63 | 0.93 | 0.13 |
| *Trifolium pratensis* | 0.07 | 0.10 | 0 | 0 |
| *Trifolium repens* | 5.13 | 26.06 | 2.13 | 5.53 |
| *Vicia sepium* | 0.20 | 0 | 0.24 | 0 |
| *Vicia cracca* | 0.02 | 0 | 0.02 | 0 |
| *Veronica filiformis* | 0.53 | 0.03 | 0.77 | 0.07 |
| *Veronica chamaedrys* | 2.21 | 0.03 | 0.81 | 0 |
| *Hedera helix* | 0.40 | 0 | 0.26 | 0.02 |
| *Galium verum* | 0 | 0 | 0.02 | 0 |
| *Leontodon autumnalis* | 0.03 | 0.15 | 0 | 0 |
| *Lotus corniculatus* | 0.35 | 0 | 0 | 0 |
| *Calystegia sepium* | 0 | 0 | 0.05 | 0 |
| *Primula vulgaris* | 0.02 | 0 | 0 | 0 |
| *Aegopodium podagraria* | 0.05 | 0 | 0 | 0 |
| *Anthriscus sylvestris* | 0.19 | 0 | 0.04 | 0 |
| *Hypericum maculatum* | 0.02 | 0 | 0 | 0 |
| *Glechoma hederacea* | 0.20 | 0 | 0 | 0 |
| *Filipendula ulmaria* | 0 | 0 | 0.27 | 0 |
| *Potentilla erecta* | 0 | 0 | 0.01 | 0 |
| *Malva sylvestris* | 0 | 0 | 0 | 0.03 |
| *Lamium album* | 0.28 | 0 | 0 | 0 |
| **Non-insect pollinated forbs** |  |  |  |  |
| *Bellis perennis* | 0.01 | 0.36 | 0.41 | 0.33 |
| *Cerastium fontanum* | 0.31 | 0.55 | 0.37 | 0.19 |
| *Plantago lanceolata* | 0.15 | 0.24 | 0 | 0 |
| *Plantago major* | 0.19 | 0.04 | 0.07 | 0 |
| *Rumex crispus* | 0.25 | 0.09 | 0.07 | 0.33 |
| *Rumex obtusifolius* | 1.69 | 0 | 0.33 | 0.48 |
| *Stellaria media* | 0.12 | 0.02 | 0.08 | 0.05 |
| *Taraxacum* aggregate | 0.35 | 1.87 | 0.76 | 2.29 |
| *Urtica dioica* | 5.77 | 0 | 5.37 | 0.02 |
| *Veronica persica* | 0.01 | 0 | 0 | 0 |
| *Veronica hederifolia* | 0.01 | 0 | 0 | 0 |
| *Geranium robertianum* | 0.09 | 0 | 0.13 | 0 |
| *Galium aparine* | 0.09 | 0.03 | 0.06 | 0 |
| *Lapsanna communis* | 0.12 | 0.04 | 0.10 | 0.03 |
| *Rumex acetosa* | 0.39 | 1.36 | 0.06 | 0.02 |
| *Stellaria holostea* | 0.05 | 0.05 | 0.01 | 0 |
| *Geum urbanum* | 0 | 0 | 0.05 | 0 |
| *Epilobium ciliatum* | 0.01 | 0 | 0 | 0 |
| **Graminoids** |  |  |  |  |
| *Agrostis capillaris* | 3.23 | 6.97 | 1.01 | 1.14 |
| *Agrostis stolonifera* | 25.39 | 9.50 | 28.67 | 19.23 |
| *Alopecurus pratensis* | 0 | 0 | 0 | 0.03 |
| *Anthoxanthum odoratum* | 0.13 | 0.27 | 0 | 0 |
| *Arrhenatherum elatius* | 0 | 0 | 0.07 | 0 |
| *Cynosuros cristatus* | 7.80 | 3.02 | 7.58 | 6.65 |
| *Dactylis glomerata* | 3.17 | 0.86 | 6.37 | 0.67 |
| *Elymus repens* | 1.47 | 0.73 | 2.79 | 0.23 |
| *Festuca rubra* | 0.40 | 4.03 | 0.47 | 0.33 |
| *Holcus lanata* | 10.94 | 10.01 | 7.74 | 2.96 |
| *Holcus mollis* | 0.50 | 0.07 | 0.03 | 0 |
| *Lolium multiflorum* | 0.37 | 0.10 | 0.01 | 0 |
| *Lolium perenne* | 16.91 | 26.59 | 19.11 | 55.57 |
| *Phleum pratensis* | 0 | 0.03 | 0 | 0.01 |
| *Poa pratensis* | 0.01 | 0.03 | 0.11 | 0.13 |
| *Juncus articulatus* | 0 | 0.03 | 0 | 0 |
| *Juncus effusus* | 0 | 0.23 | 0 | 0 |
| *Luzula campestris* | 0.03 | 0.04 | 0 | 0 |
| *Carex flacca* | 0.03 | 0.01 | 0 | 0 |
| *Carex hirta* | 0.05 | 0 | 0 | 0 |

The principal pollen vector for each species was ascertained using Grime et al. (2007) and the BIOFLOR database (Klotz et al., 2002).
