# Supplementary material for: Organic Farming and Landscape Structure: Effects on Insect-Pollinated Plant Diversity in Intensively Managed Grasslands
Source: PLoS One. 2012 May 30;7(5):e38073. doi: 10.1371/journal.pone.0038073 (PMC3364189; doi:10.1371/journal.pone.0038073)
Supplement: Table S3 — Relationships between soil parameters and farm type (organic/conventional), position in the field (edge/centre) and the interaction between farm type and edge/centre. (DOC) [file pone.0038073.s004.doc]

Table S3: Relationships between soil parameters and farm type (organic/conventional), position in the field (edge/centre) and the interaction between farm type and edge/centre.

|  | Farm type | | Edge/centre | | Farm type x edge/centre | |
| --- | --- | --- | --- | --- | --- | --- |
| Soil parameters | t_d.f_. | P | t_d.f_. | P | t_d.f_. | P |
| Phosphorus (mg/l) | -2.459_9_ | 0.036 | --- | N.S. | 2.553_58_ | 0.013 |
| Potassium (mg/l) | --- | N.S. | 4.948_59_ | <0.001 | --- | N.S. |
| Magnesium (mg/l) | -3.148_9_ | 0.008 | --- | N.S. | 3.630_58_ | 0.001 |
| pH | --- | N.S. | 3.558_59_ | <0.001 | --- | N.S. |

Note: N.S = not significant. Analysed using Linear Mixed Effects Models. Potassium and pH were square root transformed and Phosphorus and Magnesium were log transformed to achieve normality.
