# Supplementary material for: Organic Farming and Landscape Structure: Effects on Insect-Pollinated Plant Diversity in Intensively Managed Grasslands
Source: PLoS One. 2012 May 30;7(5):e38073. doi: 10.1371/journal.pone.0038073 (PMC3364189; doi:10.1371/journal.pone.0038073)
Supplement: Table S4 — Mean ± standard error hedgerow area, hedgerow length and number of connections between hedgerows within 1 km radii of organic and conventional farms. (DOC) [file pone.0038073.s005.doc]

Table S4: Mean ± standard error hedgerow area, hedgerow length and number of connections between hedgerows within 1km radii of organic and conventional farms.

|  | Organic | Conventional |
| --- | --- | --- |
| Hedgerow parameter | Mean ± SE | Mean ± SE |
| Area (%) | 4 ± 0.321 | 4 ± 0.444 |
| Length (km) | 5 ± 0.518 | 6 ± 0.616 |
| Number of connections | 27 ± 3.445 | 36 ± 4.185 |
